# Injurious Effect of Soap Dentifrice

**Published:** 1898-02

**Authors:** 


### Injurious Effect of Soap Dentifrice.

In a discussion of Dr. McWhinney's paper on bacteriology, before the "Odontographic Society of Chicago," Dr. E. L. Clifford, D. D. S., said:

"Dr. McWhinney spoke in his paper about the bacteria of pus, and I would like to ask the members of this society to look in the mouths of their patients for the chromogenic bacteria that is around the gingival margins of the teeth, spoken of by many dentists as the brick dust or red variety. I would like to have the members of the society for a year count the number of patients they find with the red variety of chromogenic bacteria on the margins of the teeth, and I would like to have them ask these patients whether they are in the habit of using, or have used in the past, any kind of soap as a dentrifice. The chromogenic bacteria are attracting a little attention and some ideas have been advanced that we would like to prove or refute."

It has been known for some time that soap dentrifices was absolutely injurious to the teeth; those containing a great amount of sugar are also objectionable. F. S. C.

